# Unseen shadows: exploring the impacts of stalking on female college students’ lives, health, and relationships in Sindh

**DOI:** 10.3389/fpsyg.2025.1553021

**Published:** 2025-06-13

**Authors:** Abdul Hadi, Yaser Snoubar

**Affiliations:** ^1^Department of Sociology, Faculty of Arts and Sciences, Harran University, Şanlıurfa, Türkiye; ^2^Social Sciences Department (Social Work Program), College of Arts and Sciences, Qatar University, Doha, Qatar

**Keywords:** SDG 5: gender equality, SDG 10: reduced inequalities, SDG 16: peace, justice and strong institutions; stalking, violence against women, psychological impacts, interpretative phenomenological analysis

## Abstract

**Introduction:**

Stalking is a serious and often overlooked form of gender-based violence that disproportionately affects adolescent girls. In Pakistan, especially in Sindh, the issue remains under-researched, despite legal reforms aimed at criminalizing such behaviors.

**Methods:**

This study employed Interpretative Phenomenological Analysis (IPA) to explore the lived experiences of 20 adolescent female stalking victims aged 15–17 at a co-educational college in Sindh. Data were collected through semi-structured interviews.

**Results:**

Two major themes emerged: (1) the victim-stalker relationship and stalkers’ motivations, and (2) the psychological, social, academic, and financial impacts of stalking. Most stalkers were strangers who sought unwanted intimacy. The victims experienced fear, social isolation, stigma, academic disruption, and in some cases, long-term psychological trauma including hallucinations and clinical depression. Cultural norms and institutional apathy further silenced victims and exacerbated their vulnerability.

**Discussion:**

Findings highlight the urgent need for gender-sensitive training for formal institutions, public awareness campaigns, and victim-centered interventions. Policymakers must recognize stalking as both a criminal and public health concern, particularly in patriarchal settings where girls’ autonomy is routinely compromised.

## Introduction

Although stalking is not a new phenomenon, it has been recognized as a crime in the recent past. Initially, in 1990, California made stalking a criminal offense following high-profile stalking cases, such as the murder of a young actress Rebecca Schaeffer by a fan-turned-stalker in 1989, as well as the subsequent murders of four women by their stalkers in Orange County, California. Within a decade, this led to similar legislation in all U. S. States and numerous other countries (Chan and Sheridan, 2020). While both men and women are victims of stalking, women are relatively more victimized, as Smith et al. (2022) revealed that women experience stalking victimization at a rate three times higher than men. Home Office (2011) showed that stalking is predominantly a gendered crime, with over 80% of victims being women and more than 70% of stalkers being men, highlighting a trend where men tend to be the perpetrators and women tend to be the victims. This gender disparity leads researchers to frame stalking as gender-based violence rooted in societal gender inequality (Proctor, 2018). Initially, gender-based violence (GBV) was understood mainly as violence against women, who suffer most severely and disproportionately. However, definitions have expanded to include all gender identities, such as LGBTQ+ and gender minorities, who often face higher risks (Graaff, 2021; Humbert and Strid, 2024). UN Women defines GBV as harmful acts based on gender, rooted in inequality and power abuse, affecting women, men and LGBTQI+ communities.

Typically, Stalking is a form of psychological violence; however, it can take other extreme forms of violence. For instance, On March 7, 2024, a stalker named Irshad Khokhar shot law student Yusra and her brother Akash in Hyderabad. Yusra died instantly, while her brother was gravely injured. The assailant then ended his own life. Akash, Yusra’s brother, passed away at Ziauddin Hospital in Karachi on March 18. It later surfaced that she had been stalked by her killer for years, pressuring her into an intimate relationship.

Various studies indicate that female students are disproportionately victimized (Amar and Alexy, 2009; Björklund et al., 2010; Jordan et al., 2007; Wood and Stichman, 2018). For instance, research by Wood and Stichman (2018) showed that 43% of female students at a university in the upper Midwest experienced stalking at some point in their lifetime, with 13% reporting incidents during their time at the institution. In contrast to adult stalking, adolescent stalking has received less attention in research; however, studies indicate a similar pattern of victimization in adolescent females (Hare et al., 2023). For example, Fisher et al. (2014) found that nearly 20% of adolescent stalking victims are female, compared to 13.9% who are male. Additionally, most adolescent perpetrators are male. Notably, in most cases, the victim and perpetrator are acquainted with each other (Purcell et al., 2009).

Many female students in Pakistan experience sexual harassment and stalking. They face sexual harassment and stalking both on campus and in the surrounding areas. Tabassum et al. (2021) examined the prevalence of stalking among university students in Rawalpindi and Islamabad. They found that stalking is widespread, with female students being the most affected (33%), causing emotional and psychological impacts including fear, social isolation, and disruption in their education and personal lives. Unfortunately, victims often hesitate to report their victimization to formal institutions due to multiple concerns, such as loss of honor and a lack of trust in the institutions to address their complaints effectively. Despite the widespread occurrence of stalking among female students and its serious repercussions, this issue remains largely overlooked by researchers, particularly in Sindh. Here, adolescent female students frequently encounter sexual harassment and stalking on campus and in surrounding areas, where boys and men often linger outside educational institutions to harass and follow them home. This study aims to address this critical gap by conducting a qualitative investigation into stalking among female adolescent students in Sindh, thereby contributing to the existing body of literature and informing policies designed to protect vulnerable youth. This paper is the second report from our research project on stalking victimization among female college^1^ students in Sindh. In the previous study (Hadi, 2024), the first author examined the prevalence of stalking and the help-seeking behaviors of affected female adolescents using an Interpretative Phenomenological Analysis (IPA) approach. While that paper focused on the nature of victimization and victims’ responses, this study expands on those findings by exploring victim-stalker relationships, the motivations behind stalking, and the impacts on female victims. By examining the lived experiences of these victims, this study sought to illuminate the severity of the stalking issue and its detrimental effects on those affected.

### Understanding the shadows: a comprehensive review of stalking

Stalking is a form of gender-based violence that requires a robust theoretical framework to capture its structural, cultural, and intersectional dimensions. Feminist theory, with its emphasis on patriarchy as the root cause of gender-based oppression, provides a foundational lens for understanding such violence. It conceptualized patriarchy as a system comprising both structural and ideological elements (Dobash and Dobash, 1979; Hadi, 2020, 2024). The structural element denotes societal hierarchy where men hold superior status, and women have an inferior status in all institutions of society, including family, religious, legal, political, and economic. The ideological aspect emphasizes the gender-biased values and norms that uphold and justify the societal dominance of men and the subordinate status of women in all societal institutions. Together, these elements create a system of gender inequality, fostering various forms of violence against women, whether physical or non-physical. As feminist theory evolved, scholars began to recognize the limitations in its explanation based solely on gender. They argued that it is essential to acknowledge how gender interacts with other aspects of identity to create unique and compound forms of oppression and privilege.

Intersectionality theory, pioneered by Crenshaw (1991), highlighted the interconnected nature of various power systems and examines how gender intersects with other axes of inequality, such as class, race, ethnicity, and geographic location, which collectively shape women’s diverse experiences of oppression. For instance, a Black woman may face gender-based violence differently than a white woman due to the added layer of racism she experiences. Likewise, a woman from a low-income background may face distinct challenges compared to a wealthier woman, even if both experience gender-based violence.

Since the recognition of stalking as a social problem and heinous crime, researchers have carried out many studies to explore it; however, there is no consensus on its definition (e.g., Amar, 2007; Taylor-Dunn et al., 2021; Jagessar and Sheridan, 2004; Korkodeilou, 2014, 2017; Stanković, 2020). Nonetheless, the literature generally converges on three key elements: a pattern of repeated behavior, unwanted conduct that is perceived as intrusive by the victim and conduct that includes an implicit or explicit threat inducing fear in the victim (Stanković, 2020); however, this seemingly neutral framing often obscures its gendered nature. A significant legislative development in Pakistan highlights this aspect: the Pakistan Penal Code, specifically Section 354B, defines stalking as a gendered crime. According to this law, a man persistently following or trying to contact a woman to foster personal interaction, even though she has shown disinterest, or monitoring her use of the internet, email, or other forms of electronic communication, commits the offense of stalking. However, despite this legal framework, the enforcement of such laws remains inadequate, underscoring the ongoing gender power imbalances in society.

Stalkers use various strategies to stalk their targets, which commonly include a stalker showing up uninvited, waiting outside or inside buildings for the victim to arrive or leave, watching the victim from a distance, following the target during her daily and nightly routines, using telephone and sending letters, gifts, or emails (e.g., Brady, 2024; Chan and Sheridan, 2020; Jaishankar and Kosalai, 2007; Logan and Walker, 2017; Matos et al., 2019; Roberts et al., 2016; Sheridan et al., 2001; Wood and Stichman, 2018). Many of these activities may appear ordinary, harmless, and not necessarily illegal when considered individually; however, the persistence of these behaviors constitutes a real harassment campaign, which may escalate in frequency and severity and may lead to other violent acts like physical or sexual assault. This progression supports feminist arguments that stalking represents a continuum of violence rooted in gender hierarchy.

Scholars have extensively studied victim-stalker relationships and categorized them into three types: intimates or former intimates, acquaintances, and strangers (National Institute of Justice, 1996). Most research studies suggest that a stalker is usually someone the victim knows, such as a friend, coworker, classmate, or former partner (Bjerregaard, 2000; Fremouw et al., 1997; Jordan et al., 2007; Maran and Zedda, 2014). For instance, Maran and Zedda (2014) revealed that over 70% of stalking victims reported being followed by someone they knew. However, some studies found higher rates of strangers-stalkers. For example, Jaishankar and Kosalai (2007) found that 68.7% of 150 college women self-defined victims in India were not acquainted with their stalkers.

Stalking causes severe debilitating effects on the psychological well-being of victims. It not only induces fear, stress, helplessness, and terror in victims but also gives rise to many psychological problems that include anxiety, depression, suicidal ideation, and distrust that may last even years after the stalking ceased (e.g., Amar, 2007; Baum et al., 2009; Cox and Speziale, 2009; Dressing et al., 2005; Feltes et al., 2012; Fleming et al., 2013; FRA Survey, 2014; Galeazzi et al., 2009; Hare et al., 2023; Korkodeilou, 2017; Logan and Walker, 2021; Matos et al., 2019; Podaná and Imríšková, 2016; Spitzberg and Cupach, 2007; Villacampa and Pujols, 2019). Villacampa and Pujols (2019) found that most victims experienced psychological effects, including depression and panic attacks. Korkodeilou (2017) reported that stalking victimization is life-changing, with complex, long-term, and often traumatic psychosocial impacts. Additionally, it disrupts their social lives significantly, leading them to avoid certain places, change routines, modify activities, reduce social outings, disguise when going out, quit jobs or schools, take safety precautions, change cars or phone numbers and even relocate (e.g., Budd et al., 2000; Cox and Speziale, 2009; Korkodeilou, 2017; Melton, 2007; Pathe and Mullen, 1997; Purcell et al., 2002; Villacampa and Pujols, 2019). These disruptions in victims’ lives often incur substantial financial costs concerning relocation, mental health treatment, and safety measures (Brewster, 1998; Korkodeilou, 2017; Logan et al., 2006). Stalking also affects the social and interpersonal relationships of victims profoundly, and people around them may be subject to threats, harassment, or even physical violence (Logan and Walker, 2010; Sheridan et al., 2001). Consequently, they often restrict their social engagements and withdraw from their social circles to avoid embarrassment and protect their loved ones (Logan and Walker, 2009; Spitzberg, 2002), resulting in feelings of social isolation, alienation, and reduced self-esteem (Brewster, 2003; Logan and Walker, 2009). The psychological and social consequences of stalking are well documented in existing literature; however, this body of work could be strengthened by considering how intersecting forms of oppression compound the victim’s experience of violence.

When it comes to coping strategies, stalking victims use various tactics to deal with their stalkers. A victim usually ignores the stalker or restricts his access (e.g., block his phone numbers and social media accounts), alter her routines, and/or confront the stalker to demand they stop (Brady, 2024; Dutton and Winstead, 2011; Fissel, 2021; Geistman et al., 2013; Podaná and Imríšková, 2016; Tokunaga and Aune, 2017; Villacampa and Pujols, 2019; Worsley et al., 2017). These strategies often shift the burden of managing the threat onto a victim herself rather than addressing the root causes or holding a perpetrator accountable. When stalking victims seek help from others, they often prefer to seek support from family and friends rather than involving police and other formal institutions (e.g., Augustyn et al., 2020; Baum et al., 2009; Dutton and Winstead, 2011; Fisher et al., 2002; Fissel, 2021; Reyns and Englebrecht, 2014; Truman and Morgan, 2021; Villacampa and Pujols, 2019). This reluctance to involve formal systems reflects mistrust in these institutions and fear of secondary victimization. The preference for informal support networks (e.g., family and friends) highlights a widespread perception that police or judicial systems are ineffective or indifferent to gender-based violence.

### Gender inequality in Pakistan: legal frameworks and women’s plight

With a population of 245 million, Pakistan is the fifth most populous country in the world (Worldometer, 2025). The latest literacy rates reported in 2019 show a significant gap between genders, with men at 69.29% and women at 46.49% (Statistica 2024). In Pakistan’s patriarchal society, power dynamics between genders are hierarchical and unequal, with men holding a dominant status and authority in both private and public spheres. Numerous reports from various international agencies demonstrate the prevalence of gender inequality in Pakistan. The Global Gender Gap Index 2021, created by the World Economic Forum, places Pakistan as the fourth worst nation worldwide in gender equality. The Gender Inequality Index (GII), published by the UNDP in 2021, evaluated gender inequality in reproductive health, empowerment, and labor market participation, ranked Pakistan 154th out of 189 countries. The pervasive gender inequality creates a conducive environment for violence against women. The Thomson Reuters Foundation Annual Poll 2018 ranked Pakistan as the fifth most dangerous country for women experiencing domestic violence and the fourth for honor-based violence, including honor killings.

A singular portrayal of the “typical” Pakistani woman is inadequate, as women’s social status in Pakistan is not homogenous due to the intersection of gender with various other forms of exclusion. The degree and nature of oppression faced by women differ based on factors such as ethnicity, religion, social class, and whether they live in rural or urban areas. Rural regions experience slightly higher rates of gender-based violence compared to urban areas, and women without educational background face higher risks of these incidents, which decrease among women with higher educational attainment (Ferdous et al., 2017; Iqbal and Fatmi, 2021; UN Women Pakistan, 2023). According to Iqbal and Fatmi (2021), Pakistani women living in rural areas face higher rates of violence than those in cities, and women with less education are more prone to such violent experiences.

Pakistan has signed seven international human rights treaties, with four specifically focusing on gender equality: the Universal Declaration of Human Rights (UDHR), the Convention on the Elimination of All Forms of Discrimination against Women (CEDAW), the United Nations Convention on the Rights of the Child (UNCRC), and the Sustainable Development Goals (SDGs). These treaties place an obligation on member states to safeguard women’s rights. In response to these international commitments, the government of Pakistan has enacted numerous laws and policies geared toward addressing and mitigating violence against women, such as Section 509 of the Pakistan Penal Code (PPC) and the Protection Against Harassment of Women at the Workplace Act of 2010 to prevent sexual harassment. The government expanded the act in 2022 to cover not only formal workplaces but also home-based workers and other areas where women might experience harassment. Additionally, the government legislated section 354B to the Pakistan Penal Code in 2021, criminalizing stalking with imprisonment and fines. Despite the international and national commitment of the Pakistani government to combat violence against women, the services for seeking redress, support, and justice are often inaccessible or inadequate for victims. The pursuit of timely and effective justice has become a significant challenge for many women, contributing to a low reporting of violence against women, ranking Pakistan 129th out of 140 countries in the Rule of Law Index 2022.

### The purpose of study

Stalking is a serious issue that has a devastating impact on victims’ mental health and daily lives. Despite these significant effects, the issue remains largely underexplored. This study examines the lived experiences of female college students who have been victimized by stalking. More specifically, this study aimed to identify victim-stalker relationships, the motivation of stalking, and its impacts on the female victims at a co-educational college in Sindh through their lived experiences. This study had the following objectives:

1. To identify the victim-stalker relationships and the perceived motivation of stalking.
2. To examine the impact of stalking on victims’ lives.

### Importance of the study

Since this topic is under-studied in Sindh, this study enriches existing literature in various ways. First, it facilitates a comprehension of the phenomenon, especially its consequences for victims. Second, it reveals shortcomings in the criminal legal system, thereby offering suggestions to rectify it to secure victims and lead offenders to prosecution. Third, it gives a voice to stalking victims, which is crucial for enhancing public awareness and encouraging victims to seek timely help.

### Research design

This study used the Interpretative Phenomenological Analysis (IPA) approach, developed by Smith in the mid-1990s (Smith, 1994, 1996), because it offers a robust framework for exploring how individuals interpret personally significant and emotionally complex experiences, such as stalking. Given that stalking often evokes feelings of fear, vulnerability, and violations of personal space, IPA is particularly well-suited for revealing participants’ subjective lived experiences in a detailed and nuanced way. While other qualitative approaches, like grounded theory and thematic analysis, are valuable, they are less capable of providing the idiographic and interpretative depth required for this study. IPA fosters profound engagement with participants’ processes of meaning-making, enabling researchers to explore not only what these experiences mean to individuals but also how they interpret them. This approach recognizes the researcher’s role in interpreting these meanings and encourages an exploration of the deeper context that lies beyond surface-level descriptions.

## Materials

In Interpretative phenomenological analysis, semi-structured interviews are recommended as the optimal method of data collection (Willig, 2007). This study employed an interview schedule to guide each interview, incorporating prompts and follow-up questions to encourage participants to elaborate on and explore their experiences. The research included three broad questions designed to explore the understanding and experiences of stalking victims, which are:

1. Could you please tell me about your relationship with the stalkers
2. Could you please tell me about the motivation of the stalker?
3. Could you please tell me about the impact of stalking on your life?

### Participant recruitment

The study focuses on adolescent female students, a group who often experience stalking. A co-educational college in Sindh was selected for its accessibility and the feasibility of data collection on this sensitive issue, as victims of stalking are often reluctant to disclose their victimization. Before recruiting participants, the first author of this research met with a college administration to discuss my research and its objectives and obtain permission to conduct it in the institution. We are grateful to the college staff, especially the principal, for permitting us to conduct the research and provide substantial support and resources. The study’s inclusion criteria involved being a female student who self-identified as a stalking victim. Therefore, the screening questions for participants were, “Have you ever been stalked?” However, before this question, students of all classes were given information about stalking, defining it as a repeated pattern of unwanted and intrusive behavior that causes emotional distress or fear in the victim.

Researchers use small sample sizes for conducting IPA studies. Smith and Osborn (2007, 2015) argued that qualitative researchers should purposefully select samples to gain insight into the experiences under study. In this study, twenty participants were recruited using purposive sampling as the aim was not to quantify results but to gain rich, in-depth information about the participants’ experiences of stalking victimization.

### Data collection/procedure

Before data collection, a pilot study was conducted with two female adolescent stalking victims to assess the clarity and relevance of the interview questions. Although these interview data were excluded from the final analysis, they helped to refine the wording and sequence of questions to elicit more detailed, reflective responses. All participants, including students’ parents, were given adequate information about the study’s objectives and nature, and their consent was obtained. Given the sensitive and often dangerous nature of stalking, several measures were taken to safeguard the confidentiality and safety of both the interviewees and the researcher. In this context, pseudonyms were assigned to each participant. Before conducting the interviews, researchers sought permission to record the interviews and assured participants about the confidentiality of their information.

Semi-structured interviews were conducted in person to ensure privacy and minimize distractions. The interviews lasted between 40 min and an hour and involved the victims discussing and reflecting on their experiences with stalking. As the subject matter was sensitive, researchers hired a psychologist to provide individual counseling sessions to students if needed.

### Data analysis

The present study analyzed the transcribed data following the recommended guidelines for IPA by Smith et al. (2009). The analysis process utilized a case-by-case approach, thoroughly evaluating each transcript individually, followed by an integration of cases. The iterative nature of IPA necessitated constant reflection and reexamination of the transcripts and themes. The analysis comprised six stages: (1) reading and rereading transcripts, (2) notetaking, (3) developing emergent themes, (4) identifying connections across themes, (5) moving to the next case (transcript), and (6) looking for patterns across Cases.

## Findings

The study interviewed stalking victims aged 15 to 17, who provided detailed accounts of their experiences of stalking (Table 1 about the details of participants and their relationships with Stalkers). The participants offered in-depth accounts concerning the effects of stalking on their mental health, daily life, and relationships. Two main themes emerged from the interviews: The Victim-Stalker Relationship and the Motivation of the Stalkers and its impact on the victims (Table 2 about themes).

**Table 1:** Details of participants and their relationships with stalkers.

Participant (pseudonym)	Age	Relationship with stalker
Suhni	16	Stranger (Male)
Popati	15	Classmate and cousin (Male)
Ambreen	16	Stranger (Male)
Zareena	17	Relative (Male)
Durr e Shahwar	16	Stranger/collegefellow (Male)
Naseema	15	Stranger (Male)
Madiha	16	Stranger (Male)
Farhat	15	Stranger (Male)
Marvi	17	Classmate (Male)
Beenish	17	Stranger (Male)
Naila	15	Stranger (Male)
Dahshila	15	Stranger (Male)
Moomal	15	Stranger/collegefellow (Male)
Rahila	15	Stranger (Male)
Rukhsana	16	Stranger (Male)
Ayesha	16	Relative (Male)
Zahida	17	Stranger (Male)
Saba	17	Classmate (Male)
Abida	16	Stranger (Male)
Sasui	16	Stranger (Male)

**Table 2:** Key themes and supporting quotes.

Theme	Supporting quotes
Theme 1. The victim-stalker relationship and stalkers’ motivation	“The stalker, whom I did not know, sought to form friendship and relationship.”
Theme 2. Impacts of stalking on victims *Sub-theme: everyday life disruption and socio-psychological impacts.* *Sub-theme: effects on social and interpersonal relationships.*	“I always used to feel upset and unmotivated. I had a clear goal in life, but I did not know how to achieve it. My parents told me that if I wanted to continue my studies, I needed to wear a veil. They warned me that if I did not comply with their instructions, they would force me to stay at home. Consequently, I decided to follow their wishes.”
	“When I was being stalked, people around me began distancing themselves. My friends started staying away from me, causing a severe blow to my self-confidence. Despite knowing this should not have occurred, it did, leaving me feeling isolated and unsupported.”

### Theme 1. The victim-stalker relationship and stalkers’ motivation

While most studies reveal that women were often stalked by someone they knew, a few studies show that most stalkers were strangers to their targets, in this study, all identified stalkers were male. Seventy-five percent of the victims reported that their stalkers were strangers, while others indicated that the stalkers were classmates or relatives. Stranger stalkers with no affiliation to the college were estimated to be in their early twenties. In all cases, the stalkers attempted to establish intimate relationships with their targets despite knowing that their feelings were not reciprocated. They clung to the hope that their persistent behavior would eventually lead to intimacy. One of the students, Moomal, reported, “The stalker, whom I did not know, sought to form friendship and relationship.” Another participant, Naseema, stated that she was followed, coerced, and threatened to accept the offer of an intimate relationship. Additionally, while college administration responded to complaints from female students regarding stalking by male students within the institution by expelling the offenders, it refrained from taking action in cases that occurred off-campus.

He stalked me for a year without ever communicating with me. After that time, he approached me and said, "I come here for you, but you do not respond." He then handed me a piece of paper with his phone number, forcing me to call on the phone. (Suhni, 16, stranger)

He left a ring wrapped in a letter written on white paper at the entrance gate of our house. I read the letter, in which he wrote some nonsensical things like, "I love you," "Talk to me," and "Be my friend." (Durre Shahwar, 16, stranger)

These passages suggest that stalkers often perceive their behavior as an act of love rather than a criminal offense. This misconception is frequently reinforced by various poems, songs, and films—particularly in the Bollywood film industry—where stalkers are glorified and portrayed as passionate lovers. Such narratives idealize stalking as a means of initiating a romantic relationship, implying that relentless pursuit will ultimately win the affection of the targeted individual.

I informed the college principal that a boy loitered outside the college every day and harassed me. The principal advised me to hire a rickshaw and suggested that I handle my personal issues by myself. (Zahida, 17, stranger)

This quote highlights a systemic failure to recognize stalking as a serious form of gender-based violence rather than merely a private or individual issue. By framing stalking as a “personal issue,” the college administration shifted responsibility onto the victim while absolving itself of accountability. Such kind of institutional apathy reflects how patriarchal norms are entrenched within educational systems. As a result, it not only perpetuates harassment but also discourages future reporting, further strengthening a culture of impunity.

### Theme 2. Impacts of stalking on victims

This theme can be divided into two sub-themes: everyday life disruption and socio-psychological impacts and effects on social and interpersonal relationships.

#### Sub-theme: everyday life disruption and socio-psychological impacts

Stalking is a serious issue that can cause significant disruptions in the lives of its victims, forcing them to make changes in their lives, such as avoiding places where they might find stalkers, altering their appearance, changing phone numbers, and implementing additional safety measures. Stalking can also have debilitating effects on their mental well-being. Participants reported that being stalked negatively affected their daily lives, such as taking time off from college, highlighting the disruption to their academic lives. It caused them to feel fear, unpredictability, feeling insecure, and psychological distress, leading to a decline in their quality of life. 80% of the participants (16) mentioned that the traumatic experience continued to affect them even after the violence ended, causing lasting and irreparable damage to their emotional and psychological well-being. While four participants of the study disclosed that they feared their stalkers might carry out an acid attack on them, one participant shared that her stalker threatened to throw acid on her if she refused his marriage proposal. As a result of psychological distress, one victim had to seek help from a clinical psychologist to manage her psychological distress, leading to considerable financial costs.

I was frightened and did not tell anyone about my victimization. I even stopped going to college for a few days. Being stalked deeply disturbed me. Before this, I went to college alone, but afterward, I started waiting for another girl or a family member to accompany me. This experience caused me to lose my self-confidence. To this day, I am still scared of being stalked or followed again. (Beenish, 17, stranger)

The passage illustrates that the victim experienced an overpowering fear and helplessness. Her decision not to report or disclose her victimization underscores the internalized shame and the fear of not being believed or supported. Additionally, the immediate impacts on her daily life become evident as she avoids college for some time and depends on others for a sense of security. This shift in behavior signifies a clear loss of autonomy and confidence, emphasizing how stalking restricts girls’ freedom of movement and control over their own lives.

If I heard any noise or knock on the door of our house at night, I would immediately think he had come to harm me. I was so afraid that I felt unsafe even in my own home and was terrified to go outside, fearing he might be waiting for me. This fear still haunts me today. (Durr e Shahwar, 16, stranger)

This paragraph illustrates the long-term psychological effects of stalking. The victim describes an increasing sense of paranoia and hyper-vigilance—common trauma responses—as she begins to associate everyday sounds with the potential presence of her stalker. The continued fear of harm, even in her own home, emphasizes how harassment can erode a person’s sense of personal safety.

My cousin (cousin-cum-stalker) spread false rumors about me, accusing me of meeting a boy in private, which caused my friends, teachers, and classmates to distance themselves from me. These rumors caused me to fall into a deep depression and suffer from memory loss. I constantly felt threatened by my cousin's blackmail. I began experiencing hallucinations and feeling disconnected from the world. I stopped attending college, barely spoke at home, and struggled to recognize my parents due to the loss of my memory. It was a hard time for me. As a result of these traumatic events, I eventually sought counseling from a clinical psychologist. (Popati, 15, classmate and relative)

The social consequences of harassment are evident here. False rumors spread by the cousin-cum-stalker, likely grounded in patriarchal notions of honor and control over women’s conduct, have led to her social exclusion. Her withdrawal from education and communication and difficulties in recognizing her parents reflect a profound disruption in her psychological health. This situation highlights the urgent need for psychosocial support for victims, particularly in contexts where cultural norms silence their experiences.

I was intensely fearful, constantly worried that he might find me alone, throw acid on me, or touch my body. (Dahshila, 15, stranger)

Dahshila’s narrative reveals the intense fear of sexual assault and acid attack, reflecting the pervasive threat of violence experienced by girls in rigid patriarchal societies. These fears indicate how harassment operates both as abuse and as a mechanism to force girls into silence and submission.

In some cases, victims have been compelled to cover their entire bodies by wearing veils. For instance, the father of Sasui forced her to wear a veil to avoid stalking if she wanted to continue her education. He threatened that if she did not comply with his orders, he would force her to leave school. As a result, she began wearing a veil, suggesting the deprivation of her right to choose her attire style. Another victim, Nadia, resorted to covering her face to protect herself from unwanted attention from men. Such lifestyle changes demonstrate the profound impact of stalking on a person’s choice of dress and lifestyle.

His behavior was terrifying, causing me great distress. He would constantly harass me, leaving me trembling as I made my way home. I was afraid that he might physically harm me or give me a letter which, if seen by my parents, would force me to leave college. I still feel fear; confronting him was hard. After that incident, I did not dress up well or use cosmetics because I believe that girls’ dress and makeup provoke males to harass and stalk. I would keep cosmetics in my purse and use them in college. (Nadia, 16, stranger)

The narrative of Nadia reveals the intense emotional toll of being stalked, characterized by feelings of fear, anxiety, and a pervasive sense of vulnerability. Her trembling and the fear of physical harm or social consequences—such as being forced to leave college—underscore how stalking operates as a form of psychological terror that threatens not only her physical safety but also her educational goals and family stability. The fear that the letter could damage her family’s honor leads her parents to force her to abandon college, highlighting the cultural and familial pressures on students. This threat to the family’s reputation adds to the student’s trauma. This illustrates the intersection of gender and societal expectations regarding female behavior, where victimization is viewed as shameful. Her subsequent withdrawal from makeup and dressing up indicates an effort to regulate her appearance and avoid further harassment. This shift in behavior illustrates an internalized victim-blaming mentality, implying that she associates cosmetic use with attracting male attention. Nadia’s belief that a girl’s appearance invites harassment points to deeply ingrained societal gender norms that hold women accountable for male violence.

#### Sub-theme: effects on social and interpersonal relationships

Stalking can have a persistent and chronic impact on the social and interpersonal relationships of victims. This study identified that 70% of victims reported being misunderstood, excluded, and left isolated by their friends and colleagues while going through the ordeal of being stalked. Some participants in the study expressed their grievances that when a girl or woman experiences stalking, she is often misperceived and held responsible for her victimization by people around her. Instead of helping a victim, friends, teachers, and classmates are more likely to point fingers at the victim’s character and leave her alone to face the stalker.

The institution doubted me and created the impression that I was the type of girl who attracted stalkers. My cousin refused to accompany me, and there were judging eyes around me. (Abida, 16, stranger)

My classmate spread rumors about me, saying that I had a loose moral. As a result, my friends and teachers began to distance themselves from me and believed that I was a "bad girl". One teacher, who was once very close to me, even stopped talking to me. (Saba, 17, classmate)

These paragraphs highlight the complex ways in which gender, cultural norms, and institutional responses intersect to shape the experiences of adolescent girls. The intersectionality of these factors weaves a complex web of vulnerability and silence that can prevent female victims from disclosing, seeking help, or feeling validated in their experiences of victimization. The victimization extends beyond the personal safety of victims to social perception of their character and moral values. The fear of being viewed as morally loose, shameful, loss of honor, or untrustworthy can have long-lasting impacts on the mental well-being of victims.

## Discussion

The aim of this study was to gain a rich and detailed understanding of the stalking victimization of female college students in Sindh. Specifically, the study sought to explore the victim-stalker relationship, the stalkers’ motivation, and stalking impacts on the victims by interviewing twenty participants with a semi-structured interview guide and analyzing the data through interpretative phenomenological analysis.

The findings diverge from much of the existing literature on adolescent and adult stalking, which highlights relational dynamics—where victims are commonly targeted by classmates, peers, or acquaintances (Fisher et al., 2014; Fremouw et al., 1997; Haugaard and Seri, 2003; Hare et al., 2023; Jordan et al., 2007; Matos et al., 2019; Purcell et al., 2009). Even within the Pakistani context, Tabassum et al. (2021) found that 60% of female victims in Rawalpindi and Islamabad were stalked by ex-friends, reinforcing global patterns. However, this study challenges the prevailing narrative by revealing that most participants identified their stalkers as strangers with no connection to their college. It marks a significant departure from the relational framing prevalent in Western and some local studies, highlighting the importance of an intersectional approach that considers how region, mobility, gender norms, and access to public space shape stalking experiences. Notably, the findings align with research from India (Jaishankar and Kosalai, 2007), where stranger stalking is prevalent. However, the divergence in results highlights the importance of avoiding a one-size-fits-all approach to stalking experiences and emphasizes the need for context-sensitive, intersectional analyses of gendered violence.

This study emphasizes the long-lasting emotional and psychological effects of stalking on victims, revealing that most victims continued to endure the trauma of victimization long after the stalking had ended. This result aligns with the existing literature (Baum et al., 2009; Cox and Speziale, 2009; Korkodeilou, 2017; Logan and Walker, 2010; Matos et al., 2019; Melton, 2007; Pathe and Mullen, 1997; Podaná and Imríšková, 2016; Sheridan et al., 2001; Taylor-Dunn et al., 2021; Villacampa and Pujols, 2019), which highlights the enduring repercussions of stalking victimization. It challenges the conventional understanding of victimization as a temporary experience, underlining the long-term nature of recovery and the insidious ways harassment-related trauma can persist. This finding underscores the significance of recognizing stalking as not just a criminal offense but a public health problem, calling for early intervention and continuous support to victims in terms of counseling and legal help. It suggests the need for broader support structures that go beyond addressing the immediate impact of stalking to provide long-term psychological care for victims. Additionally, this study emphasized the necessity of public awareness campaigns to reshape societal values, reduce stigma, and encourage victims to seek timely help.

A novel finding of the study is an alarming prevalence of fear of acid attacks among victims, with four of them reporting concerns that their stalkers might resort to acid attacks as revenge for the rejection of their proposals and advances. One victim reported receiving an explicit threat of acid attack from her stalker if she refused his advances substantiated this fear It reflects a broader, deeply entrenched culture of violence geared toward controlling the autonomy and choices of girls and women. Such threats call for the urgent need to address acid violence, which has long been a concern in countries including India, Pakistan, Bangladesh, Nepal, Uganda, and Cambodia (Acid Survivors Trust International, 2015). While some acid attacks are perpetrated by women, this does not alter the fact that this violence is grounded in gender inequalities, and they may be committing it within a system where such violence is normalized or where they themselves experience abuse and violence. The high-profile case of an adolescent, Laxmi Agarwal, who was attacked by a family ‘friend’ after rejecting his romantic advances (Times of India, 2005), highlights the issue of acid attacks as a means of retribution for the rejection of male advances in India. The National Crime Records Bureau (2018) reports around 200 acid attack cases annually, further evidencing the widespread nature of the violence in India. Similarly, hundreds of women experience similar violence each year in Pakistan (Yousaf and Purkayastha, 2016). According to ActionAid (2017), acid attacks are frequently directed at women who reject romantic advances of males in Bangladesh (ActionAid, 2017). These findings are crucial in understanding how the threat of acid violence intersects with the gendered dynamics of stalking and harassment. The fear of acid attacks manifests the cultural and structural conditions that allow men to maintain control over the bodies and choices of women, particularly in patriarchal societies where the autonomy of women is constantly policed.

This study highlights how victims alter their way of living to avoid receiving unwanted attention from male harassers and stalkers. These changes included avoiding usual activities, reducing social activities, taking days off college, and changing phone numbers. This finding supports the previous studies (Baum et al., 2009; Dressing et al., 2005; Korkodeilou, 2017; Matos et al., 2019; Melton, 2007; Pathe and Mullen, 1997; Purcell et al., 2002; Sheridan et al., 2001), which similarly document the disruption of lives of victims as they attempt to regain a sense of control. While these adaptations may offer temporary relief, they emphasize a critical issue, such as the loss of autonomy of girls and the emotional toll of altering their lifestyles, highlighting the power dynamics inherent in stalking. Additionally, this study contributes to the existing literature by offering a novel insight as some victims began to wear less makeup, change their attire style, and start wearing veils because of the belief that wearing ‘inappropriate’ attire and makeup could incite men to stalk and sexually harass them. This change reflects and reinforces a social narrative that places the responsibility for stalking on women. Unfortunately, some women internalize this belief and blame the girls and women for sexual harassment or sexual assault, as one of the victims of the study held girls and women accountable for stalking because of their “inappropriate” dressing and makeup. This self-blame approach reflects a broader societal problem where women are frequently loaded with unwarranted guilt, exacerbating their psychological distress.

The finding also highlights the profound impacts of stalking on victims’ social relationships and financial stability. Unlike previous studies that highlight the disintegration of relational ties because victims distanced themselves from their loved ones in a bid to protect them from the stalkers (Logan and Walker, 2009; Spitzberg, 2002), this study shows that relationship deterioration occurs due to stigmatization and social exclusion of victims as well as victim-blaming attitudes of people around the victims. It reveals a broader societal issue where, instead of providing support, people often re-victimize victims by shifting the blame onto them. The social exclusion and secondary victimization show the deep-rooted societal norms that penalize victims rather than perpetrators, thereby further entrenching the marginalization of victims. One victim reported seeking counseling from a clinical psychologist to get over the trauma and psychological distress. Such circumstances call for more comprehensive public education and community-based interventions that challenge victim-blaming, reduce stigmatization, and foster a more supportive environment for victims. Another consequence of stalking is the financial burden placed on victims, as some participants reported frequently changing their phone numbers and using autorickshaws for transportation to college.

This study points to a lack of efficient support systems and legal protection that might allow victims to reclaim their autonomy. These inefficiencies reveal a critical gap in both prevention and intervention programs, which inadequately address broader systemic factors, such as gender-based violence and social stigmatization. We connect this issue to the broader gender equality agenda outlined in the 5th Sustainable Development Goal (SDG 5), which emphasizes empowering women and girls, ending gender-based violence, and ensuring equal opportunities in all aspects of life. In Sindh, gender inequality manifests through various forms, including gender-based violence and limited access to education and employment for women. By linking our findings to SDG 5, we highlight the necessity of addressing these issues not only to align with global goals but also to promote social cohesion, economic development, and democratic citizenship at the local level.

## Conclusion

The research offers crucial findings that enhance the existing body of knowledge about stalking victimization among girls and women. Contrary to much of the existing literature, which indicates that stalking victims often know their perpetrators, this study reveals that most female students were stalked by strangers, with just a small percentage being targeted by classmates or relatives.

The research offers crucial findings that enhance the existing body of knowledge about stalking victimization among girls and women. Contrary to much of the existing literature, which indicates that stalking victims often know their perpetrators, this study reveals that most female students were stalked by strangers, with just a low percentage of them targeted by classmates or relatives.

The study shows how victims of stalking experience long-lasting psychological and social impacts, with some expressing fears of violent retaliation, such as acid attacks, which exacerbated their psychological distress. This fear is substantiated as, in one case, a victim received direct threats from her stalker. It is not just a personal injury to the victims but also a violation of women’s rights to equality and freedom. The findings also emphasize the lifestyle changes that victim made to avoid their stalkers, including reduced social interactions, absences from college, and alterations to their daily routines, personal attire, and phone numbers. It profoundly affects the academic lives of victims, compelling them to alter their daily routines and lifestyles. Some victims adopted veils and modified their clothing to evade unwanted attention.

Despite the legislation of section 354B to the Pakistan Penal Code in 2021, which criminalizes stalking with imprisonment and fines, stalking remains a significant issue for girls and women due to ineffective enforcement. One of the main challenges is that many formal actors lack specific training on the newly enacted laws and have limited gender-sensitive and victim-centric attitudes. The Police and justice sectors must undergo enhanced training and focus on increasing female representation. Additionally, strategies must be developed that empower victims, hold perpetrators accountable, and reshape societal attitudes toward harassment and stalking.

### Limitations and strengths of the study

This study was conducted in a college setting to investigate and characterize the nature of stalking and its effects on victims. It is crucial to note that the findings cannot be universally applied to all women who experience stalking, as the study was not intended for statistical generalization. Nonetheless, the study sheds light on the prevalence of stalking among female students and highlights its significant impact on their lives. Moreover, it gives voice to stalking victims and contributes to raising public awareness by highlighting stalking as a form of gender-based violence and advocating for institutional and community-based protective measures.

## Data Availability

The original contributions presented in the study are included in the article/supplementary material, further inquiries can be directed to the corresponding author.

## Footnotes

^1^In Pakistan, "college" refers to higher secondary education, which precedes university-level studies. It typically encompasses Grades 11 and 12, culminating in the Higher Secondary Certificate (HSC), after which students may pursue undergraduate degrees at a university.
